# Knowledge and attitudes towards sexually transmitted infections and their associated factors among late adolescents in Kota Bharu, Kelantan: A cross-sectional study

**DOI:** 10.51866/oa.1173

**Published:** 2026-09-29

**Authors:** Shahrizam Tahir, Aida Maziha Zainudin, Nur Suhaila Idris

**Affiliations:** 1 Department of Family Medicine, School of Medical Sciences, Universiti Sains Malaysia, Kubang Kerian, Kelantan, Malaysia.; 2 Department of Pharmacology, School of Medical Sciences, Universiti Sains Malaysia, Kubang Kerian, Kelantan, Malaysia.

**Keywords:** Sexually transmitted infections, Health knowledge and attitude, Adolescent, Religion, Malaysia

## Abstract

**Introduction:**

Sexually transmitted infections (STIs) represent a significant health challenge, particularly for late adolescents in conservative regions including Kota Bharu, Kelantan, where cultural norms may influence health literacy. This study aimed to determine the levels of knowledge and attitudes towards STIs and identify their associated factors among late adolescents in Kota Bharu.

**Methods:**

A cross-sectional study was conducted among 228 late adolescents (aged 18–21 years) from four higher-educational institutions. A validated bilingual online questionnaire was used to assess sociodemographic characteristics, high-risk behaviours, religiosity (Malay version of the Duke University Religiosity Index) and knowledge and attitudes towards STIs. Multiple linear regression (MLR) was utilised to identify independent predictors of attitudes.

**Results:**

A total of 228 completed responses were analysed. Most participants were women (68.9%) and Muslim (84.2%). The mean knowledge score was 15.15/26 (standard deviation [SD]=5.17), with the human immunodeficiency virus awareness score being high (94.7%); significant gaps existed regarding other STIs. The mean attitude score was 57.10/65 (SD=6.51), indicating a generally protective mindset. MLR identified four independent factors significantly associated with positive attitudes: higher knowledge level (adjusted b=0.616, P<0.001), non-organisational religious activity (adjusted b=0.679, P=0.010), non-Malay ethnicity (adjusted b=–3.205, P=0.002) and maternal primary educational level (adjusted b=–4.085, P=0.021).

**Conclusion:**

While late adolescents possess protective values, their functional knowledge of STIs remains superficial. Knowledge and internalised religious practices may contribute to positive attitudes. Targeted culturally sensitive health education and adolescent-friendly primary care services are essential to improve reproductive health outcomes.

## Introduction

Sexually transmitted infections (STIs) present a major global disease burden, affecting nearly 1 million people daily.^1^ Despite being among the most manageable and preventable diseases, STI cases keep rising both in developed nations, such as the United States and Germany, and developing nations, such as Malaysia.^1,3^ There are over 30 distinct organisms, including bacteria, viruses, fungi and parasites, known to cause STIs.^4^ Examples of STIs include human immunodeficiency virus (HIV) infection, syphilis, chlamydia, gonorrhoea and hepatitis B.^3^ Although STIs are primarily transmitted via close, unprotected sexual contact, non-sexual routes such as blood transfusion, mother-to-child transmission and needle sharing can also spread such infections.^3,5^

Late adolescents include individuals aged 18–21 years.^6^ Late adolescence is a critical period of development, marked by increasing autonomy and psychological maturity. This is a phase of transition into adulthood.^5^ According to the National Health and Morbidity Survey in Malaysia, 7.3% of adolescents have engaged in sexual activities.^3^ Early sexual debut and lack of awareness regarding the consequences of unprotected sex significantly predispose late adolescents to STIs.^7^ Raising morbidity burden among adolescents leads to the increasing utilisation of national healthcare resources.^1^

Reports have shown a rising rate of STIs among adolescents in Malaysia.^8^ This can be attributed to a generally low level of awareness and attitude regarding STIs among Malaysian youth.^9^ The rising trend of STIs significantly increases the risk of unwanted complications such as infertility, pelvic inflammatory disease and even death.^10^ STIs also pose a risk of detrimental mental health effects such as anxiety and depression, which negatively affect patients’ quality of life.^11^ This can be due to guilt, marginalisation and stigma around the issue.

The National Strategic Plan for Sexual and Reproductive Health recommends increasing awareness and knowledge among youth as one of the key pillars in reducing the rising incidence of STIs among the targeted age group.^12^ Although multiple studies have assessed the level of knowledge among adolescents,^9,10,13,14^ there is still a paucity of data focusing on late adolescents in Kota Bharu, Kelantan. The conservative cultural context of Kota Bharu, Kelantan, potentially limits sexual health awareness among late adolescents. Previous studies have frequently overlooked how sociocultural and spiritual beliefs influence sexual health knowledge and attitudes. This study moves beyond traditional health literacy by deconstructing religiosity into multiple domains to evaluate its distinct impacts on reproductive health mindsets. Understanding how these religious dimensions intersect with clinical awareness is essential for family care providers aiming to design localised, culturally appropriate primary care interventions. This calls for localised research, which is essential for developing effective culturally appropriate reproductive health policies.

This study aimed to assess the levels of knowledge and attitudes towards STIs among late adolescents in Kota Bharu, Kelantan. Additionally, it aimed to identify whether sociodemographic factors, religiosity, high-risk behaviours and knowledge level are associated with attitudes towards STIs among this population.

## Methods

This cross-sectional study was conducted from July to December 2025. Participants were recruited from four randomly selected institutions around the urban area of Kota Bharu, Kelantan, namely Universiti Malaysia Kelantan, Universiti Teknologi MARA Kampus Kota Bharu, Kolej PolyTech MARA and Politeknik Kota Bharu. Late adolescents aged 18–21 years enrolled in the target tertiary institutions were included. The study population was in accordance with the age limits set by the American Academy of Pediatrics.^6^ Individuals who were diagnosed with mental disorders were excluded.

Participants were selected via multistage institutional selection followed by convenience sampling via digital platforms. After approval was obtained from the ethics committee of Universiti Sains Malaysia, we began liaising with each institution’s student affairs. We then approached respective student leaders to provide a link and QR code for the online questionnaire. The questionnaires were then distributed through students’ social media and online messaging platforms, such as WhatsApp, Telegram and email, to collect data. Students who agreed to participate were asked to provide their consent prior to completing the survey. Institutional identifiers were not included in the questionnaire to protect student privacy and encourage honest responses on sensitive sexual health topics across campus environments.

Ethical approval was obtained from the Human Research Ethics Committee, Universiti Sains Malaysia (Protocol: USM/JEPeM/KK/24040330). Permission to conduct the research among students at higher institutions was also granted by the Ministry of Higher Education. Each response was stored in a password-protected database, which was accessible only to the research team.

The study instrument was a self-administered questionnaire, converted into an online Google Form to facilitate distribution and accessibility, comprising four domains. The first domain evaluated the sociodemographic and high-risk behaviour profiles of participants. The sociodemographic data collected included age, sex, ethnicity, religion, parental education and household income based on the Department of Statistics Malaysia categories.^15^ High-risk behaviours included smoking/vaping, substance abuse and pornography use. The second domain assessed participants’ religiosity using the Malay version of the Duke University Religiosity Index (DUREL-M). This 5-item scale measures organisational religious activity (ORA), non-organisational religious activity (NORA) and intrinsic religiosity (IR). It has previously been translated and validated in Bahasa Malaysia.^16,17^ The DUREL-M previously demonstrated good parallel reliability (0.70) and test—retest reliability (Spearman’s rho=0.68, P<0.01) among Malaysian nursing students.^16^ The third domain assessed the level of knowledge towards STIs using a questionnaire adapted from the regional literature.^7^ This 26-item questionnaire was modified from the International AIDS Questionnaire-English Version.^18^ It evaluates participants’ knowledge on the causes, transmission and complications of STIs. Correct answers are scored 1, while incorrect or unsure answers are scored 0. The fourth domain explored attitudes towards STIs using an adapted 13-item tool on a 5-point Likert scale: *strongly disagree, disagree, neutral, agree* and *strongly agree J* Questions 2 and 7 are negatively marked. This tool assesses perceptions of screening, condom efficacy and health- seeking behaviour.^7^ Higher scores indicate a more protective and positive attitude towards STIs. The questionnaire was designed in English and translated into Bahasa Malaysia, suitable for the local dialect. Permission to use this questionnaire was obtained from the developers.

The sample size was calculated for each objective, with the largest requirement derived from Objective 3, which sought to identify the factors associated with attitudes. With a standard deviation (SD) of 4.8 from the previous literature and a detectable difference of 2, the minimum sample size required was 182.^19^ After a 20% non-response rate was accounted for, the final target sample size was 228.

Data were analysed using IBM SPSS Statistics version 29.0, International Business Corporation (IBM), Armonk, New York, United States. Descriptive statistics, including means, SDs, frequencies and percentages, were utilised for the sociodemographic characteristics and levels of knowledge and attitudes. Inferential analysis was conducted to determine the factors associated with the total attitude score as the dependent variable following a two-step approach. We employed simple linear regression (SLR) to screen the independent variables, including the sociodemographic characteristics, religiosity and knowledge level. Variables with P<0.25 in the SLR or those deemed clinically significant were entered into stepwise multiple linear regression (MLR). Model fitness was assessed using the ANOVA F-test, with P<0.05 considered statistically significant.

## Results

### Sociodemographic characteristics

A total of 228 valid fully completed questionnaires were retrieved during the data collection period and included in the final analysis. The mean participant age was 19.21 years (SD=0.96), with an age range of 18–21 years. The cohort was predominantly women (n=157; 68.9%), Malay (n=184; 80.7%) and Muslim (n=192; 84.2%). These sociodemographic outcomes reflected the local demographic composition of Kota Bharu, Kelantan.

Regarding socioeconomic background, 44.3% of the participants belonged to the B40 income category (<RM 3030). The majority self-reported a close relationship with their parents (n=217; 95.2%) and lived in on-campus student accommodation (n=188; 82.5%). In terms of relationship status, 95.6% (n=218) were single, while only 4.4% (n=10) reported being in an intimate relationship.

### High-risk behaviours

The prevalence of high-risk behaviours was notably low within the cohort. Only 4.4% (n=10) identified themselves as smokers or vapers, and the use of illicit substances was negligible at 0.4% (n=1). Furthermore, 7.5% (n=17) of the participants admitted to having used or been exposed to pornographic materials. Regarding clinical history, only 0.9% (n=2) disclosed a personal history of an STI, and 3.1% (n=7) reported knowing someone with an STI.

### Religiosity

Table 1 shows that the participants exhibited a high level of religiosity. The mean total score for all domains, including ORA, NORA and IR, was 22.40 (SD=3.85) out of 27. Religious engagement showed the highest score within the IR domain (mean=13.98, SD=2.09), followed by NORA (mean=4.55, SD=1.57).

**Table 1:** Descriptive statistics of the religiosity domains in the DUREL among the participants (N=228).

Domain	Mean (SD)	Min, max
Total DUREL	22.40 (3.852)	5, 27
DUREL domain 1: organisational religious activity	3.87 (1.324)	1, 6
DUREL domain 2: non-organisational religious activity	4.55 (1.574)	1, 6
DUREL domain 3+4+5: intrinsic religiosity	13.98 (2.091)	3, 15

DUREL: Duke University Religiosity Index, SD: standard deviation.

### Knowledge of STIs

The mean knowledge score was 15.15 (SD=5.17), with a score range of 1–26. The majority of the participants identified HIV infection as an STI (n=216; 94.7%). However, the level of knowledge regarding other specific infections was significantly lower. For example, only 32.9% (n=75) and 47.8% (n=109) correctly identified chlamydia and gonorrhoea as STIs, respectively. Misconceptions regarding non-STIs were also present. Tuberculosis and asthma were incorrectly identified as STIs by 5.7% (n=13) and 1.8% (n=4) of the participants, representing overall incorrect or uncertain rates of 33.8% and 5.3%, respectively. Misconceptions regarding casual transmission persisted, with only 57.9% (n=132) correctly identifying that sharing food, drinks or clothes does not spread STIs. Additionally, 25.4% (n=58) of the participants answered incorrectly or were unsure when asked if mosquito bites transmit STIs. Furthermore, knowledge regarding long-term complications was poor: Only 12.3% (n=28) were aware that STIs could lead to ectopic pregnancy, and 26.8% (n=61) identified cervical cancer as a potential complication. Table 2 presents the participants’ knowledge of STIs.

**Table 2:** Knowledge scores regarding sexually transmitted infections among the participants (N=228).

Bil.	Item	Answer, n (%		Response, n (%)	
		Yes	No	Unsure	Correct	Incorrect
1.	HIV infection and AIDS are examples of sexually transmitted infections (STIs).	216 (94.7)	5 (2.2)	7 (3.1)	216 (94.7)	12 (5.3)
2.	Gonorrhoea is an example of a STI.	109 (47.8)	16 (7.0)	103 (45.2)	109 (47.8)	119 (52.2)
3.	Syphilis is an example of a STI.	135 (59.2)	9 (3.9)	84 (36.8)	135 (59.2)	93 (40.8)
4.	Tuberculosis is an example of a STI.	13 (5.7)	151 (66.2)	64 (28.1)	151 (66.2)	77 (33.8)
5.	Chlamydia is an example of a STI.	75 (32.9)	22 (9.6)	131 (57.5)	75 (32.9)	153 (67.1)
6.	Asthma is an example of a STI.	4 (1.8)	216 (94.7)	8 (3.5)	216 (94.7)	12 (5.3)
7.	STIs are caused by bacterial infections.	120 (52.6)	59 (25.9)	49 (21.5)	120 (52.6)	108 (47.4)
8.	STIs are caused by viruses.	159 (69.7)	24 (10.5)	45 (19.7)	159 (69.7)	69 (30.3)
9.	Mosquito bites can spread STIs.	4 (1.8)	170 (74.6)	54 (23.7)	170 (74.6)	58 (25.4)
10.	Having multiple unprotected sexual partners can increase the risk of contracting STIs.	220 (96.5)	2 (0.9)	6 (2.6)	220 (96.5)	8 (3.5)
11.	History of receiving blood/blood product donations can spread STIs.	124(54.4)	53 (23.2)	51(22.4)	124(54.4)	104(45.6)
12.	Sharing syringe needles increases the risk of spreading STIs.	170(74.6)	21 (9.2)	37(16.2)	170(74.6)	58 (25.4)
13.	Sharing foods, drinks, clothes and toilet increases the risk of spreading STIs.	46 (20.2)	132(57.9)	50(21.9)	132(57.9)	96 (42.1)
14.	Hugging and shaking hands increase the risk of spreading STIs.	12 (5.3)	190(83.3)	26(11.4)	190(83.3)	38 (16.7)
15.	A mother with a STI can spread her disease to her newborn during delivery.	108(47.4)	41 (18.0)	79(34.6)	108(47.4)	120(52.6)
16.	Taking contraceptive pills can reduce the risk of acquiring STIs.	27 (11.8)	97 (42.5)	104(45.6)	97 (42.5)	131(57.5)
17.	Using male condoms can reduce the risk of acquiring STIs.	134 (58.8)	31 (13.6)	63 (27.6)	134 (58.8)	94 (41.2)
18.	Monogamy/faithfulness to one’s sexual partner can reduce the risk of spreading STIs.	176 77.2)	9 (3.9)	43 (18.9)	176 (77.2)	52 (22.8)
19.	Not engaging in multiple sexual partners is an effective method to avoid contracting STIs.	195 (85.5)	9 (3.9)	24 (10.5)	195 (85.5)	33 (14.5)
20.	Genital ulcers are a sign that a person may have a STI.	111 (48.7)	15 (6.6)	102 (44.7)	111 (48.7)	117 (51.3)
21.	Pain during urination is a sign that a person may have a STI.	112 (49.1)	27 (11.8)	89 (39.0)	112 (49.1)	116 (50.9)
22.	Genital discharge is a sign of a STI.	85 (37.3)	43 (18.9)	100 (43.9)	85 (37.3)	143 (62.7)
23.	Can a person with a STI show no symptoms?	93 (40.8)	41 (18.0)	94 (41.2)	93 (40.8)	135 (59.2)
24.	Infertility and miscarriage are among the complications of STIs.	67 (29.4)	48 (21.1)	113 (49.6)	67 (29.4)	161 (70.6)
25.	Cervical cancer is a complication of a STI.	61 (26.8)	71 (31.1)	96 (42.1)	61 (26.8)	167 (73.2)
26.	Ectopic pregnancy is a complication of a STI.	28 (12.3)	84 (36.8)	116 (50.9)	28 (12.3)	200 (87.7)

HIV: human immunodeficiency virus.

### Attitudes towards STIs

The participants’ attitudes towards STIs were generally positive and protective. The mean total attitude score was 57.10 (SD=6.51), with a score range of 36–65. The highest agreement was observed for the necessity of premarital screening (92.6% agreed or strongly agreed; mean=4.74, SD=0.68) and the intention to seek immediate medical treatment if symptomatic (mean=4.68, SD=0.65). Table 3 demonstrates the participants’ attitudes towards STIs.

**Table 3:** Attitude scores towards STIs among the participants (N=228).

Bil.	Item	Response, n (%)					
		Strongly disagree (1)	Disagree (2)	Neutral (3)	Agree (4)	Strongly agree (5)	Mean (SD)
1.	I believe that condom use is effective in preventing the transmission of STIs.	18 (7.9)	13 (5.7)	70 (30.7)	60 (26.3)	67 (29.4)	3.64 (1.189)
2.	I believe that if both partners are already infected with an STI, condom use is no longer necessary during sexual intercourse.^*^	(5) 125 (54.8)	(4) 33 (14.5)	(3) 50 (21.9)	(2) 9 (3.9)	(1) 11 (4.8)	4.11 (1.164)
3.	I believe that the prohibition of prostitution can curb the spread of STIs.	12 (5.3)	1 (0.40)	18 (7.9)	29 (12.7)	168 (73.7)	4.49 (1.035)
4.	Screening for STIs is an effective intervention for public health.	11 (4.8)	2 (0.9)	24 (10.5)	33 (14.5)	158 (69.3)	4.43 (1.045)
5.	Premarital screening for STIs is essential.	2 (0.9)	2 (0.9)	13 (5.7)	20 (8.8)	191 (83.8)	4.74 (0.684)
6.	The consumption of sexually explicit materials may contribute to high-risk sexual behaviours.	6 (2.6)	7 (3.1)	33 (14.5)	37 (16.2)	145 (63.6)	4.35 (1.011)
7.	STIs are not dangerous because they are treatable.^*^	(5) 137 (60.1)	(4) 38 (16.7)	(3) 38 (16.7)	(2) 5 (2.2)	(1) 10 (4.4)	4.26 (1.090)
8.	If left untreated, STIs such as HIV infection can be fatal.	3 (1.3)	4 (1.8)	23 (10.1)	30 (13.2)	168 (73.7)	4.56 (0.845)
9.	I would be deeply concerned if I were to be diagnosed with an STI.	11 (4.8)	9 (3.9)	32 (14.0)	25 (11.0)	151 (66.2)	4.30 (1.145)
10.	I am concerned that engaging in casual sex increases my risk of contracting an STI.	8 (3.5)	1 (0.4)	19 (8.3)	28 (12.3)	172 (75.4)	4.56 (0.930)
11.	I am concerned about contracting an STI if I engage in unprotected sexual intercourse.	6 (2.6)	3 (1.3)	39 (17.1)	42 (18.4)	138 (60.5)	4.33 (0.981)
12.	I must seek immediate medical treatment if I exhibit symptoms of an STI.	0 (0)	1 (0.4)	21 (9.2)	27 (11.8)	179 (78.5)	4.68 (0.655)
13.	I will advise my sexual partner to seek immediate medical treatment if they exhibit symptoms of an STI.	3 (1.3)	0 (0)	21 (9.2)	22 (9.6)	182 (79.8)	4.67 (0.753)

* Questions 2 and 7 are reverse-scored items. SD: standard deviation, STI: sexually transmitted infection.

### Factors associated with attitudes towards STIs

The SLR analysis identified several factors significantly associated with the attitude scores: age (P=0.009), ethnicity (P=0.002), religion (P=0.004), maternal primary educational level (P=0.017), NORA (P=0.001), IR (P=0.018), overall religiosity (P=0.042) and total knowledge score (P<0.001). Table 4 shows the SLR analysis of the independent factors associated with attitudes towards STIs.

**Table 4:** SLR analysis of the factors associated with attitudes towards STIs among the participants (N=228).

Variable	SLR		
	b (95% CI)	t	P
**Sociodemographic characteristics**			
Age	1.174 (0.296 to 2.053)	2.633	0.009
Ethnicity Malay Non-Malay	Ref -3.364 (-5.477 to -1.250)	-3.136	0.002
Household income <RM 3030 RM 3030-6619 >RM 6619	Ref -1.103 (-3.157 to 0.951) 0.926 (-0.871 to 2.724)	-1.058 1.015	0.291 0.311
Sex Female Male	Ref -0.760 (-2.597 to 1.077)	-0.815	0.416
Religion Muslim Non-Muslim	Ref -3.385 (-5.679 to -1.091)	-2.908	0.004
Marital status Single Married	Ref 4.921 (-7.956 to 17.798)	0.753	0.452
Place of living In-campus Out-campus	Ref 0.090 (-2.150 to 2.330)	0.079	0.937
Relationship with parent Good Poor	Ref 0.467 (-3.508 to 4.443)	0.232	0.817
Maternal educational level No formal education Primary education Secondary education Higher education	Ref -5.021 (-9.129 to -0.913) 0.354 (-1.396 to 2.103) 0.518 (-1.200 to 2.235)	-2.408 0.399 0.594	0.017 0.691 0.553
Paternal educational level No formal education Primary education Secondary education Higher education	Ref -1.730 (-5.699 to 2.240) -0.231 (-2.031 to 1.570) 0.736 (-1.005 to 2.476)	-0.859 -0.252 0.833	0.391 0.801 0.406
Currently in a relationship Yes No	Ref -1.254 (-5.411 to 2.903)	-0.549	0.553
**Sociodemographic characteristics**			
History of drug/illicit substance abuse Yes No	Ref 1.106 (-11.786 to 13.998)	0.169	0.866
Smoking/vaping status Smoker Non-smoker	Ref 1.151 (-3.006 to 5.309)	0.546	0.586
Use of pornographic materials Yes No	Ref -2.052 (-5.284 to 1.180)	-1.251	0.212
Personal history of contracting a STI Yes No	Ref -1.412 (-10.547 to 7.723)	-0.304	0.761
Know someone who has/had a STI Yes No	Ref -2.844 (-7.768 to 2.081)	-1.138	0.256
**Religiosity**			
DUREL domain 1: organisational religious activity	-0.261 (-0.905 to 0.383)	-0.799	0.425
DUREL domain 2: non-organisational religious activity	0.884 (0.354 to 1.414)	3.288	0.001
DUREL domain 3+4+5: intrinsic religiosity	0.487 (0.084 to 0.890)	2.380	0.018
TOTAL DUREL	0.228 (0.008 to 0.448)	2.045	0.042
**Knowledge**			
Level of knowledge of STIs	0.571 (0.424 to 0.719)	7.646	<0.001

CI: confidence interval, DUREL: Duke University Religiosity Index, SLR: simple linear regression.

The significant independent variables obtained from the SLR analysis were then included in the MLR analysis. Four independent factors were significantly associated with the attitude score, as shown in Table 5. Knowledge showed the strongest positive association with attitude, with every 1-point increase in the knowledge score resulting in a 0.616-point increase in the attitude score. Private religious activity (NORA) was also independently associated with a higher attitude score. Conversely, both non-Malay ethnicity and maternal primary educational level were associated with lower attitude scores among the participants.

**Table 5:** MLR analysis ofthe factors associated with attitudes towards STIs among the participants (N=228).

Variable	MLR		
	Adjusted b (95% CI)	t	P
Ethnicity Malay Non-Malay	Ref -3.205 (-5.252 to -1.158)	-3.086	0.002
Maternal educational level No formal education Primary education	Ref -4.085 (-7.553 to -0.616)	-2.321	0.021
**Religiosity**			
DUREL domain 2: non-organisa- tional religious activity	0.679 (0.167 to 1.191)	2.615	0.010
Knowledge towards STIs	0.616 (0.477 to 0.755)	8.725	<0.001

CI: confidence interval, DUREL: Duke University Religiosity Index, MLR: multiple linear regression, STI: sexually transmitted infection.

## Discussion

### Key findings

This study revealed that the late adolescents in Kota Bharu, Kelantan, possessed a mean score of 15.15 out of 26 for their level of knowledge regarding STIs. Comparably, a similar study among university students in the central zone of Malaysia showed a mean knowledge score of 23.3 (SD=8.4).^7^ This also resonates with a finding from a previous study among 1075 Malaysian young adults, which demonstrated moderate knowledge regarding STIs, particularly HIV infection and AIDS.^9^ Despite this, the late adolescents demonstrated highly positive and protective attitudes towards STI prevention and management (mean=57.10/65). The MLR identified that higher knowledge levels (adjusted b=0.616, P<0.001) and internalised religious practices (NORA) (adjusted b=0.679, P=0.010) were significantly associated with higher attitude scores, while non- Malay ethnicity (adjusted b=–3.205, P=0.002) and maternal primary educational level (adjusted b=–4.085, P=0.021) were associated with lower attitude scores.

### Knowledge gaps and misconceptions

While HIV/AIDS awareness was high (94.7%), the recognition of other common STIs such as chlamydia (32.9%) and gonorrhoea (47.8%) was substantially lower. This heavily HIV-centric awareness mirrors the findings of Folasayo et al. among university students in Central Malaysia.^7^ Similarly, a study assessing the knowledge level among Turkish adolescents showed high awareness towards HIV/AIDS, but the majority failed to recognise other STIs.^20^ Such findings suggest that national health campaigns may be focusing heavily on HIV and related illnesses while unintentionally neglecting other prevalent bacterial STIs. These findings underscore a critical gap in sexual health education in Malaysia. Although officially integrated into the national curriculum via the Reproductive and Social Health Education (PEERS) module, its delivery is hindered by teacher discomfort, a lack of specialised training and cultural taboos. Consequently, instruction often lacks depth, omitting crucial details regarding transmission pathways, symptoms and complications. This systemic instructional gap explains why late adolescents enter higher education with a superficial HIV-centric awareness while remaining largely oblivious to other common STIs.^21^

Furthermore, this study revealed persistent misconceptions regarding the mode of transmission of STIs, including sharing of food, drinks and toilets; hugging; and mosquito bites. Several studies across multiple regions worldwide have reported similar findings on transmission misconceptions.^9,10^ In contrast, youths in Gondar, Ethiopia, have shown fewer misconceptions regarding STI transmissibility as a result of effective health promotion.^22^ In the current study, the participants also demonstrated a poor understanding of long-term complications such as ectopic pregnancy (12.3%). This signals a lack of functional sexual health literacy. Moreover, most participants were unaware of the asymptomatic nature of STIs (n=135; 59.2%). This is particularly concerning in primary care, as a lack of knowledge regarding the asymptomatic nature is a major barrier to timely treatment-seeking.^23^

### Attitudes and influence of religiosity

The generally positive attitudes observed in this cohort likely reflect the conservative socioreligious landscape of Kelantan. The majority of the Muslim community views the act ofpre-marital sexual activities as sinful and as something that should be avoided.^3^ The present analysis indicated that religiosity, specifically NORA, was more strongly associated with positive attitudes than public religious gatherings. This suggests that internalised faith and personal moral commitment act as a protective buffer against high-risk perceptions. This resonates with local reports indicating that religious values are significant protective factors against permissive sexual attitudes among Malaysian students.^19^ Therefore, primary healthcare providers must integrate education that respects local cultural values when delivering sexual health education.

### Sociodemographic factors associated with attitudes

The study identified ethnicity and maternal education as factors significantly associated with the attitude scores. The non-Malay participants scored significantly lower on the attitude scale than their Malay counterparts (adjusted b=–3.205, P=0.002). This disparity may be due to the integration of health messages with religious values within the majority of the Malay—Muslim community. Comparably, a study among 1171 university students in Malaysia showed that non- Malays were more permissive to pre-marital sexual relationships (P<0.001).^24^

Additionally, a notable finding was that the adolescents whose mothers had primary education exhibited poorer attitudes than those whose mothers had no formal education (adjusted b=– 4.085, P=0.021). Because specific family communication dynamics, parental health literacy and underlying value orientations were not directly measured in this study, these subgroup variations remain exploratory observations. Further research utilising qualitative or targeted measures is needed to explore how family background and health communication shape reproductive health- seeking behaviours among diverse adolescent populations.

### Implications for family medicine

Primary care providers serve as the first points of contact for late adolescents. Given the gaps identified, there is a critical need for comprehensive education, including empowering preventive measures. Education, including promoting condom use among adolescents, is an effective way to promote sexual health.^25^ Additionally, clinicians should incorporate sexual health counselling beyond traditional barrier methods to include prevention strategies such as vaccinations, HIV prophylaxis and emerging evidence-based interventions including doxycycline post-exposure prophylaxis (DoxyPEP) for eligible individuals. Furthermore, primary care providers should take a proactive step by establishing adolescent-friendly services.^26^ Ensuring stigma-free environments with guaranteed confidentiality to overcome the shame and stigma associated with sexual health in conservative regions is also essential. Moreover, active parental involvement should be pursued. Primary care providers can act as mediators to navigate through areas of sensitive sexual health issues. Open communication between young adults in the family helps create a safe and stigmafree environment.^27^

### Strengths and limitations

To the best of our knowledge, this study is the first to explore the determinants of STIs among late adolescents, specifically on the east coast of Malaysia, addressing a critical data gap. The use of validated instruments, such as the DUREL-M, ensures theoretical and empirical reliability. However, several limitations must be noted. The cross-sectional design prevents the establishment of causal relationships. The use of convenience sampling and an online questionnaire may have introduced selection and social desirability biases, as the participants might have overestimated their religious adherence or positive attitudes to align with social norms. The recruitment pattern led to unadjusted clustering effects, with the participants sharing homogeneous social and religious views. Additionally, our adapted study instrument did not evaluate the adolescents’ literacy regarding contemporary prevention modalities such as vaccinations, HIV prevention or DoxyPEP. Future research should include these modern prevention strategies to capture the evolving landscape of reproductive health. Furthermore, because the survey utilised an open voluntary digital link, the total number of students who viewed the invitation but declined to participate or left the form incomplete could not be tracked, preventing the calculation of the true response rate. The findings may not also be generalisable to youth in Kelantan, particularly those who do not pursue higher education or have limited internet access.

## Conclusion

In conclusion, this study provides critical insights into STI-related knowledge and attitudes among late adolescents in higher-educational institutions in Kota Bharu, Kelantan. We discovered that while the late adolescents held a predominantly positive attitude towards STIs, their functional knowledge remained superficial. Significant gaps were identified regarding recognising common infections beyond HIV infection, their asymptomatic nature and their severe long-term complications. The primary factors independently associated with a protective attitude were higher knowledge levels and internalised religious practices (NORA). Conversely, lower attitude scores were observed among the non-Malay participants and those whose mothers had primary education. These findings highlight the need for inclusive and appropriate multicultural health communication. They also imply that late adolescents in Kelantan require comprehensive and culturally sensitive sexual health education beyond basic HIV awareness. Furthermore, primary care providers should take proactive steps to establish adolescent-friendly, stigma-free services and involve parents as key mediators to improve health literacy and treatment-seeking behaviour in this conservative landscape.

## Data sharing statement

The raw data utilised in this study can be obtained from the corresponding author upon reasonable request.

**How does this paper make a difference in general practice?**

- This study provides localised insights into knowledge and attitudes towards STIs among late adolescents in the unique underexplored sociocultural landscape of Kelantan.
- It identifies critical gaps in knowledge regarding non-human immunodeficiency virus infections and complications, helping primary care doctors design targeted counselling.
- It highlights internalised religiosity (non-organisational religious activity), ethnicity and maternal education as predictors of protective attitudes, enabling the integration of culturally suitable sexual health education.
- It advocates for active parental involvement and confidential adolescent-friendly services to overcome barriers of shame and stigma in conservative regions.
